# Factors affecting the implementation of the Health Extension Workers Programme in Kavango East Region, Namibia

**DOI:** 10.4102/hsag.v31i0.3245

**Published:** 2026-04-10

**Authors:** Dionisius M. Nghikevali, Daniel O. Ashipala, Julia Amadhila

**Affiliations:** 1 Department of General Nursing Sciences, Faculty of Health Sciences and Veterinary Medicine, University of Namibia, Rundu, Namibia; 2 Department of General Nursing Sciences, Faculty of Health Sciences and Veterinary Medicine, University of Namibia, Oshakati, Namibia

**Keywords:** delivery of health care, factors, health extension worker, implementation, Namibian Health Extension Workers Programme

## Abstract

**Background:**

The Namibian Health Extension Workers Programme (HEWP) plays a crucial role in ensuring equitable access to healthcare, particularly in rural areas. Little research exists on the factors affecting the implementation of HEWP; however, there is an urgent need for these services.

**Aim:**

To explore and describe the factors affecting the implementation of the HEWP in Rundu District, Kavango East Region, Namibia.

**Setting:**

The study was conducted at the five primary health care clinics in the Rundu District, Kavango East region of Namibia.

**Methods:**

An exploratory descriptive qualitative research design was employed. The population consisted of 28 health extension workers (HEWs). Data saturation was reached with 13 participants; two more participants were added, bringing the total to 15, and no new information emerged. Data were collected through semi-structured interviews using an audiotape recorder and field notes before being analysed thematically using Braun and Clarke’s six steps of data analysis. Trustworthiness was ensured using Lincoln and Guba’s four criteria. Ethical approval was obtained from the School of Nursing Research Committee, with the principles of beneficence, autonomy, justice and non-maleficence being adhered to.

**Results:**

Three prominent themes emerged: Organisational and resource-related factors affecting HEWP implementation; HEWs’ capacity and engagement; and recommendations to ensure the effective implementation of the HEWP.

**Conclusion:**

The findings revealed that there are several barriers that need to be overcome before the desired outcome of HEWP can be successful. Major obstacles to programme implementation include insufficient resources, excessive staff turnover and inadequate training.

**Contribution:**

The results of this study can be used to develop targeted interventions and strategies to mitigate the factors encountered during the implementation of the HEWP in Namibia and similar settings.

## Introduction

Ensuring equitable access to healthcare is a universal, complex, multidimensional health challenge, especially in rural areas. According to Coombs, Campbell and Caringi (2022), hard-to-reach areas have fewer healthcare providers, which impairs rural health systems and their ability to function effectively. The development and implementation of rural health services to reach distant communities is thus one of the main goals of economic growth and development of most countries worldwide (Aynalem & Melesse 2021). According to Gizaw, Astale and Kassie (2022), one of the best ways to improve quality health services in rural communities is through the implementation of the Health Extension Workers Programme (HEWP). Desta et al. (2017) defined health extension workers (HEWs) as professional healthcare service providers who work in health centres in rural and medically underserved areas, responsible for delivering integrated preventive, promotive, and basic curative health services. Health extension workers are trained with the aim of extending health services to communities far from health facilities, promoting preventive healthcare practices towards communities, undertaking community outreach activities, and visiting houses to provide communities with knowledge and skills on important health-related topics (Tefera 2022). Health extension workers are increasingly being recognised as an integral component of the health workforce, who are needed to achieve and promote public health goals – especially in low- and middle-income countries (Schaaf et al. 2020). Health extension workers are used to deliver and dispense health care services to patients with a broad range of health issues (Medhanyie et al. 2012).

The implementation of HEWPs has been shown to have a significant impact worldwide. A study conducted in Ethiopia by Alam, Tasneem and Oliveras (2012) found that the introduction of HEWPs has led to improvements in maternal and child health, hygiene, sanitation and community awareness regarding health, as well as a decrease in communicable diseases. Gebretsadik, Melaku and Haji (2020) added that HEWs have also contributed to a significant improvement in the utilisation of family planning, the prevention of and testing for human immunodeficiency virus (HIV), antenatal care and postnatal check-ups. Health extension workers have also been responsible for expanded vaccination services, malaria control and prevention, and the treatment of dysentery, malaria, intestinal parasites and other ailments (Afari et al. 2014). Additionally, Perry & Hodgins (2021) reported that HEWs can promote a holistic approach to a patient’s well-being and play a crucial role in rendering first aid to patients at the primary care level.

Numerous developing countries have a challenge with overburdened health facilities. Most facilities are understaffed, and many people walk long distances to access health services, hence the need for HEWPs. A health extension programme was first implemented in Ethiopia in 2003, aiming to provide health services that reduce preventable diseases, lower maternal and child mortality rates, promote health services and refer critical cases to a higher level of care (Schaaf et al. 2020). In recent years, HEWs have improved healthcare needs in the most remote communities of the United States (Hartzler et al. 2018). Health Extension Workers Programmes have been widely utilised in various countries for several years and are considered crucial for rendering appropriate healthcare in rural areas (Asmamaw et al. 2023). According to Méllo et al. (2023), HEWPs are mostly found in low- and middle-income countries, including 18 African countries, 12 Asian countries, and five Latin American countries.

Yitayal et al. (2014) noted that HEWPs were established to help countries meet their Millennium Development Goals (MDGs). The expansion of these programmes is a core component of creating broader health systems and one of the strategies regarded as necessary to achieve universal primary health care (PHC) among rural populations (Kare, Gujo & Yote 2021). Health extension workers are organised to spend 75% of their time visiting families by walking house to house and performing outreach activities in communities, and 25% of their time at a health post providing contraceptives and family health services (Salgedo, Ayele & Abriham 2020).

In Namibia, the HEWP was introduced in 2010, but programme activities were only implemented in 2012. The aim of the programme was to extend the provision of health services to the most remote communities in all 14 regions (Ministry of Health and Social Services [MoHSS] 2022). According to the MoHSS (2022), in some communities in Namibia, the distances between health facilities and communities are too long; hence, the need for HEWs. The government thus recommended that all communities located more than 10 km away from health facilities with a population of more than 5000 people should be provided with health extension services. As no research has been conducted on this phenomenon in Namibia, this study aimed to explore and describe the factors affecting the implementation of the HEWP in Rundu District, Kavango East Region, Namibia. Findings from this study can be used to develop ongoing, targeted strategies and interventions to mitigate the barriers identified.

## Research methods and design

A qualitative exploratory, descriptive and contextual design was executed to explore and describe the factors affecting the implementation of the HEWP in Rundu District, Kavango East Region, in Namibia. A qualitative approach explores how people make sense of their surroundings, experiences and understanding of a phenomenon (Polit & Beck 2021). The qualitative exploratory and contextual design thus allowed for an in-depth exploration and understanding of HEWs regarding the factors affecting the implementation of the HEWP in Rundu District, Kavango East Region, Namibia. This approach enabled the researcher to explore individuals’ understanding of factors affecting the implementation of HEWP (Corcoran, Baum & Dunn 2024). The qualitative approach enabled a comprehensive exploration of the interviewees’ perceptions, lived experiences and challenges, utilising non-numerical analyses to understand the complexity of their roles and interactions within the community and health system (Brink, Van der Walt & Van Rensburg 2018).

### Setting

The study was conducted in the Rundu District of the Kavango East Region, Namibia, where the HEWP is implemented. This district, characterised by a diverse population and unique healthcare challenges such as the remoteness of PHC facilities and limited resources, provided a rich context for assessing factors influencing the programme’s implementation. The study specifically involved five PHC clinics, namely, Kayengona, Shambyu, Baramasoni, Ncaute, and Takvase, with a total of 28 HEWs actively delivering essential health services. These clinics, located in peri-urban settings, play a critical role in promoting health education and preventing disease at the community level. The healthcare infrastructure in Rundu, including its referral systems and community engagement programmes, offered a comprehensive setting for examining the factors affecting the HEWP, facilitating appropriate data collection without substantial financial expenditure.

### Population and sampling

The study’s population included all 28 HEWs employed in PHC facilities in Rundu District. The sample was conveniently selected according to the following criteria: employed as an HEW, working for the MoHSS, employed for 5 years or more, and able and willing to take part. These individuals were considered well-informed and able to provide rich information that was pertinent to the study’s objectives (Polit & Beck 2021). Primary health care supervisors and the community health nurses who supervised the HEWs helped by identifying and notifying suitable interviewees about the study. They further provided a list of HEWs who met the inclusion criteria and were prepared to be interviewed, along with their contact details. All interviews were held at the participants’ workplaces once written informed consent had been obtained. A total of 15 HEWs took part in the interviews, with data collection continuing until saturation was reached at the 12th participant, that is, no new information was being shared. This ensured that the researcher had a full understanding of what factors affect the HEWP execution in the district.

### Data collection

Data were collected through semi-structured interviews conducted between February and June 2024, with a second researcher conducting all interviews. The interviews were conducted using an interview schedule created in accordance with the study’s objectives and the existing literature. The primary language used during the face-to-face interviews was English, as all the interviewees could understand and communicate effectively in it due to their academic and professional experiences. The researcher did not have any existing relationships with the interviewees that could have influenced their motivation to take part. To ensure there was no coercion, participants were informed of the study’s purpose, the voluntary nature of their participation, and their right to withdraw or decline to answer any questions at any time. Each participant voluntarily signed an informed consent form before taking part. Confidentiality and anonymity were thoroughly described to the interviewees before the interviews, and they were assured that their data would be stored securely and used solely for this study. The data were anonymised by replacing direct identifiers like names with consistent pseudonyms or generic descriptors (e.g. ‘P’). Each interview took 30 min – 40 min, depending on the depth of the replies.

The interviews were held in locations chosen by the participants, which were often close to the clinics or community outreach areas where they worked. These settings were quiet and suitable for open conversations with no interruptions. Each interview was audio-recorded with the participants’ consent, with field notes being taken to note down behaviours, non-verbal cues and relevant environmental observations. The interviews were scheduled at convenient times for the interviewees, typically when they were not in the field, to minimise disruptions. The interviews were conducted by the second author, while the first author provided supervision and reviewed the overall process with a view to enhancing the validity of the research findings. During data collection, the second author led the interviews and took thorough field notes. The data were independently coded and analysed by the second author and an independent coder. The first author reviewed the coding and analysis process, and the three then met to reach a consensus on the final themes. Both the first author and the second author contributed to reporting and verifying the final results to ensure rigour and consistency.

The key open-ended questions posed were:

- *What factors could be affecting the implementation of the HEWP in communities in Rundu District, Kavango East Region, Namibia?*
- *What recommendations can be made to support the implementation of the HEWP in communities in Rundu District, Kavango East Region, Namibia?*

### Data analysis

The audio recordings were transcribed verbatim by the second author before being analysed using Braun and Clarke’s (2019) six steps of data analysis as cited by Ashipala and Shapopi (2022): (1) familiarisation with the data by rereading interview transcripts, listening to the recordings multiple times and taking notes; (2) generating initial codes to describe the content and label features relevant to the research question; (3) searching for themes across all interview transcripts by reviewing the coded data to identify similarities and overlaps; (4) reviewing potential themes by checking them against the organised extracts to ensure they accurately reflected the data; (5) defining and naming themes by summarising each theme in a few sentences to produce a coherent overall account; and (6) producing a report by writing a clear, convincing and comprehensive description of the data based on the analysis. The second author led the data analysis, together with an independent coder, and identified and agreed on the themes.

### Measures to ensure trustworthiness

The four principles framework of Lincoln and Guba (1985) was used to ensure the trustworthiness of this study. The researcher aimed to guarantee the trustworthiness of the study through confirmability, credibility, transferability and dependability. Confirmability was supported by multiple data collection methods, such as audio recordings and detailed field notes during individual in-depth interviews. This triangulation of data sources ensured that the findings were firmly grounded in participants’ experiences. Additionally, the researcher maintained an audit trail, documenting decisions and procedures throughout the study, which enabled the verification of the findings. Credibility was established by maintaining prolonged engagement with participants in the field to build rapport with participants and gain a deeper, accurate understanding of their culture and social context, spending extended time interacting with them in their workplace. Members checking further enhanced credibility by allowing participants to review and validate the findings. Member checking was done by sharing findings or data with the research participants to confirm accuracy and ensure the results authentically represent their experiences. This was done during interviews by summarising points for immediate verification, or after data collection by providing participants with transcripts, summaries, or entire reports for them to review and provide feedback. The goal was to achieve credibility and enhance the trustworthiness of the qualitative research by correcting misinterpretations or clarifying misunderstandings. Transferability was strengthened by providing thick descriptions of the research context, participants and methodology, enabling other researchers to assess the relevance of the findings to different settings. Through detailed accounts of participants’ experiences, the study offers insights into how the findings may be applied to other contexts. Dependability was ensured through the involvement of an independent co-coder who reviewed the data and findings, offering an external perspective on the analysis. A detailed account of the study design and methods was also provided, promoting transparency in the research process. Lengthy and varied engagement with the interviewees ensured the study’s confirmability, as the researcher engaged in reflexive practice throughout the data analysis process, regularly reflecting on his own biases, assumptions and preconceptions that had the potential to influence data interpretation.

### Ethical considerations

The study received approval from the School of Nursing Research and Ethics Review Committee (SoNREC) of the University of Namibia on 13 October 2023. The ethical clearance number is SoN 91/2023. Additional permission to conduct the study was granted by the MoHSS Institutional Research Review Board (reference number: 22/4/2/3). Gatekeepers of clinics in the Kavango East region provided further approval and access to participants. Written informed consent was obtained from all participants prior to their involvement. Participants were fully informed of the study’s purpose and potential benefits and voluntarily agreed to participate. Guidelines of the Declaration of Helsinki were followed, thus application of beneficence, non-maleficence, autonomy and justice. There was no risk to participants, as the study did not involve tests or human trials. While there were no direct benefits, the participants contributed to findings that may indirectly enhance the quality of patient care. Confidentiality of participants’ information was strictly maintained. All audio recordings, interview transcripts, and field notes are securely stored on a password-protected computer accessible only to the researcher and their study supervisors. In line with institutional research policy, the data will be retained for 5 years.

## Results

This study explored the factors affecting the implementation of the HEWP in Rundu District, Kavango East Region, Namibia and revealed three themes: Theme one, ‘organisational and resource-related factors affecting the HEWP implementation’, includes subthemes related to core issues affecting HEWs’ service delivery; the second theme, ‘HEWs’ capacity and engagement’, training and capacity gaps, motivation and recognition and supportive supervision and feedback mechanisms; and the third theme, ‘recommendations to ensure the effective implementation of the HEWP’, enhancing resource availability, strengthening training and mentorship, improving communication and coordination and fostering community collaboration and support. The quotes of the participants were mentioned as P1, P2, and so on. Table 1 shows the summary of the themes and subthemes that emerged from the analysis.

**TABLE 1:** Summary of themes and subthemes.

Themes	Subthemes
1. Organisational and resource-related factors affecting HEWP implementation	1.1. Resource availability 1.2. Core issues affecting health extension workers’ service delivery 1.3. Working conditions 1.4. Communication delays
2. HEWs’ capacity and engagement	2.1. Training and capacity 2.2. Motivation and recognition 2.3. Supportive supervision and feedback mechanisms
3. Recommendations to ensure the effective implementation of the HEWP	3.1. Creating opportunities 3.2. Training for motivation 3.3. Provision of educational materials 3.4. Strengthen supportive supervision 3.5. Ensure adequate supplies 3.6. Environmental protection needed 3.7. Boosting the community and health workers

HEW, health extension workers; HEWP, health extension workers programme.

### Theme 1: Organisational and resource-related factors affecting the health extension workers programme implementation

Participants described several organisational and resource-related challenges that hinder effective implementation of the HEWP. These included limited availability of resources and supplies, high workloads due to staff shortages, poor working conditions and infrastructure, and delays in communication and coordination. Such constraints were reported to negatively affect service delivery and overall programme performance.

#### Subtheme 1.1: Resource availability

Participants in this study identified transportation as a major challenge. A number of them reported that limited transport options and poor road infrastructure hinder their ability to reach remote areas and provide timely healthcare services. Participants described their concerns about the lack of essential resources and materials. Participants expressed concerns regarding the lack of essential resources and materials, citing these as core issues affecting HEWS’ service delivery and working conditions. The lack of these resources not only hampers the ability of HEWs to perform their duties effectively, but also affects the quality of care provided to the community:

‘For us we give first aid, for instance if I get a case for malaria … automatically I have to refer to the hospital but transport is always a problem to find. I have to call the ambulance from Rundu to pick up that person and they can tell you that there is no car or not picking up the call.’ (P12, male, 43 years)

‘We don’t have reporting tools and medicine on time; sometimes we make copies taking money from our pockets or to look for help from nearest school to make copies for reporting papers. I also don’t have a blood pressure machine.’ (P13, male, 39 years old)

#### Subtheme 1.2: Core issues affecting health extension workers’ service delivery

The interviewees highlighted the critical challenges they face in their daily operations, such as a lack of in-service training, limited knowledge and skills, a lack of opportunities for further studies, no staff incentives, poor morale, promotion and cultural factors:

‘Some of us we are not trained well, like malaria test now they use four drops but we use two, some colleagues never do any training but work in communities, parents sometimes ignore health promotion because we tell same things all time.’ (P2, female, 43 years old)

‘We don’t get promotion, and sometimes we can’t go for study leave. So, the government must give us leave for further study. It makes work very hard.’ (P13, male, 39 years old)

‘Some people in the community don’t listen to us because of their tradition and religion. They think our work is not important.’ (P8, female, 41 years old)

#### Subtheme 1.3: Working conditions

The study participants reported that the challenging working conditions they encountered significantly impacted their ability to deliver quality healthcare services. They highlighted several key issues related to their working environment, such as poor working conditions, long distances, physical strain, environmental challenges, and a lack of support and supervision:

‘Our working conditions are very hard. We walk long distances, get tired, face bad weather, and sometimes we don’t get enough support or supervision.’ (P3, female, 39 years old)

‘Our supervisors should have supportive supervision to correct us to do better; they do support but not consistently. We lack strong supervision in the field, we work far from each other, no one to ask when you don’t understand, supervisors take long to come, this year I did not see anyone here. Our supervisors they only blame us for whatever we have done wrong or faults but they don’t really adequately support us.’ (P13, male, 39 years old)

#### Subtheme 1.4: Communication delays

Participants strongly expressed that the communication challenges they encounter hinder their ability to deliver effective healthcare services. They mentioned several key issues related to communication delays, such as a lack of cell phone network coverage and limited airtime:

‘Sometimes there is no network in the village; you try to call supervisor but cannot reach. Even airtime we don’t have all the time, so it takes long to get help and patients suffer more.’ (P7, female, 36 years old)

### Theme 2: Health extension workers’ capacity and engagement

Participants discussed factors influencing their capacity and level of engagement in implementing the HEWP. Key issues included gaps in training and skills development, low motivation and recognition, and inadequate supportive supervision and feedback mechanisms. These factors were reported to affect HEWs’ confidence, performance and commitment to service delivery.

#### Subtheme 2.1: Training and capacity

This theme reflects participants’ views on training opportunities. Health extension workers highlighted that the lack of adequate in-service training has limited them from updating their skills and knowledge, particularly affecting the quality care they render to clients:

‘Lack of Inservice training for example some of us we use two drops of buffer to test malaria, we were not taught that they are changed to four but we were not trained about that.’ (P2, female, 43 years old)

‘When we usually give health promotion about HIV, patients use to request that they want to be tested by us and we were not trained to test patients for HIV in the community.’ (P6, male, 50 years old)

‘Some of our colleagues they never undergone most of the training but some did like for malaria and some never get this training but they work in different communities with no skills of testing malaria.’ (P15, female, 44 years old)

#### Subtheme 2.2: Motivation and recognition

Participants in this study expressed job dissatisfaction and lack of motivation because of the absence of incentives or rewards for their efforts:

‘Another challenge is that there are no promotions, we are just health assistants, you know in life you need to progress working for nine years with no promotion is not good.’ (P4, male, 38 years old)

‘Since we were recruited our salary never get increased and, no promotion nothing like senior and junior health extension workers.’ (P10, female, 51 years old)

‘Imagine a person working for ten years, you need to be motivated, there is no strong motivation like further studies just to improve or promotion. The ministry is not promoting us, this thing demoralizes the person.’ (P13, male 39 years old)

#### Subtheme 2.3: Supportive supervision and feedback mechanism

Participants in this study reported that they receive low supervision, guidance, resources and oversight to deliver quality care to clients, which promotes professional growth and ensures quality care for the communities they serve:

‘Our supervisors must have a supportive supervision to correct us to do better, they do support but they don’t do it consecutively.’ (P13, male, 39 years old)

‘We have a lack of strong supervision to support us, correct us during field work because we work far from each other and no one to ask for help where you don’t understand. Supervisors are taking long to come, like this year I did not see any supervisor here.’ (P14, female, 44 years old)

### Theme 3: Recommendations to ensure the effective implementation of the health extension workers programme

The participants provided suggestions to improve the effectiveness of the HEWP, including offering more transportation, providing adequate training and resources, enhancing supervision, and strengthening community engagement to ensure timely and quality healthcare delivery.

#### Subtheme 3.1: Creating opportunities

The interviewees emphasised the importance of creating opportunities for the professional growth of HEW, including supporting their development and providing further education to enhance their skills and knowledge:

‘We also need the district to send us to school; we also want to become enrolled or registered nurses one day.’ (P7, female, 39 years old)

‘I am the first intake of this programme but since we were recruited there is no promotion, the government should send us for further study.’ (P8, female, 41 years old)

#### Subtheme 3.2: Training for motivation

A HEW highlighted the need for regular training to maintain their motivation and update their knowledge. They emphasised that the government should provide ongoing training, refresher courses and opportunities for further studies to ensure that HEWs remain competent and confident in delivering healthcare services:

‘The government should provide more training … new things to refresh knowledge regularly … a regular need to be sent for further studies.’ (P7, female, 39 years old)

#### Subtheme 3.3: Provision of educational materials

The participants highlighted the need for adequate educational and communication materials to support their work. Suggestions included providing tools such as mobile phones with network support and monthly airtime to facilitate effective health education and communication with the community:

‘There should be clear guidelines in the community for health promotion, and the Ministry should provide us with leaflets to educate teenagers at schools and other community members.’ (P4, male, 38 years old)

‘The Ministry should also provide phones with network support and monthly airtime to facilitate communication and health education in the community.’ (P3, female, 41 years old)

#### Subtheme 3.4: Strengthen supportive supervision

A HEW emphasised the need for structured and consistent supportive supervision to ensure the effectiveness of the HEWP. In addition, strengthening supervision was seen as essential for maintaining accountability, improving service quality and fostering professional growth among HEWs:

‘Our supervisors must provide consistent and supportive supervision to help us improve. The supervisor should be specifically assigned to this programme, rather than a nurse with other tasks, so that challenges can be addressed promptly.’ (P13, male, 39 years old)

#### Subtheme 3.5: Ensure adequate supplies

The participants emphasised the importance of ensuring a consistent and sufficient supply of essential medications and resources. Adequate stock, including medications for conditions such as malaria and cholera, as well as proper storage containers, is critical for them to deliver timely and effective healthcare services, especially in remote areas:

‘The Ministry should provide all essential materials, including census books, daily activity books, referral books, maternal and neonatal books, and leaflet charts, so that we do not struggle while working in the community.’ (P4, male, 38 years old)

#### Subtheme 3.6: Environmental protection needed

The interviewees highlighted the need for measures to protect HEWs from challenging environmental conditions while performing their duties. Suggestions included providing shelters; protective clothing such as uniforms, raincoats, gumboots and gloves; access to safe water; and transport support for remote or bushy areas:

‘We need protection when working in bushy areas with wild animals like elephants, and shelters to keep us safe in bad weather. We also need uniforms, raincoats, gumboots, gloves, and umbrellas to work safely.’ (P10, female, 42 years old)

#### Subtheme 3.7: Boosting the community and health workers

The participants emphasised the importance of initiatives that support both them and the communities they serve. Suggestions included assigning personnel to address social issues, promoting mental health awareness, and ensuring that HEWs report and follow up on cases such as suicide or other mental health problems. One participant argued that≈strengthening collaboration between the HEWs and communities is essential for improving trust and engagement:

‘There should be someone assigned to help with social issues in the community, and health extension workers must report cases like suicide or people with mental health problems so that support can be provided on time.’ (P14, female, 44 years old)

## Discussion

This study explored the factors affecting the implementation of HEWs in Rundu District, Kavango East Region, Namibia. The findings revealed a complex interplay of systemic barriers, community transformation and strategies necessary for improving programme implementation.

One of the most significant challenges identified was transportation. The HEWs reported difficulties with long distances, poor road networks and limited transport options, which negatively affect timely referrals and access to health facilities. In emergencies, a lack of ambulances creates further delays, leaving HEWs vulnerable to community frustrations. These findings align with those of Yadeta (2020), who reported similar barriers in Ethiopia, and Kok et al. (2015), who found that transport shortages create additional burdens and erode trust. Collectively, the evidence underscores that inadequate transport remains a systemic barrier to effective service delivery in rural contexts.

Another critical challenge is the lack of resources and essential materials, including referral forms, medicines, reporting tools and medical equipment. Health extension workers often resort to using their own personal funds to make photocopies of forms or buy basic supplies. This mirrors HEWs’ experiences in Uganda, where Musoke et al. (2020) highlighted shortages of blood pressure devices and nurse bags, while Kok et al. (2015) similarly confirmed gaps in referral materials, and Getachew et al. (2021) noted low preparedness at community work due to limited resources. Such constraints directly undermine the quality and scope of care HEWs are able to provide.

Limited training and a lack of professional development opportunities also negatively affect HEWs’ motivation and performance. Participants reported limited access to in-service training, a lack of promotion prospects, and study leave. These findings align with those of the World Health Organization (WHO 2020), which emphasised that insufficient educational support weakens the professional growth of HEWs. Befekadu and Yitayal (2020) also found there to be inadequate knowledge among HEWs for managing TB and mental health. Similarly, Medhanyie et al. (2012) documented skill gaps in maternal care, while Abate et al. (2022) linked poor career development opportunities with low motivation.

Participants in this study expressed job dissatisfaction and lack of motivation because of the absence of incentives or rewards for their efforts. This finding is similar with the study conducted by Abate et al. (2022), which reported that recognition and support were significantly low among HEWs from South nations, and nationalities people’s region (SNNPR) and Oromia and were associated with low motivation. Another study consistent with the above findings highlighted that the intention of HEWs to leave was 39.5%, and the reasons to leave were low incentives and a dearth of career development opportunities (Kitila et al. 2021). According to the study by Wintrup (2023), it is also reported that HEWs were demotivated by very low salary payments compared to the expected amount and weak rewarding.

Participants indicated that traditional and religious cultural beliefs limited community acceptance of HEWs’ services, hindering effective health delivery. This aligns with WHO (2017), which reported that cultural barriers remain a persistent challenge in community health programmes.

Poor working conditions were another concern, with participants describing unsafe environments, physical strain, and minimal managerial support, all of which compromise service delivery. These findings resonate with Yadeta (2020), who reported unsafe conditions and poor institutional support for HEWs. Inadequate supervision was also noted, with oversight described as infrequent and focused on faults rather than providing support. Tesema et al. (2022) similarly argued that irregular and critical supervision reduces opportunities for skill development. A similar study by Tesema et al. (2022) reported that HEWs often experience poor supervision, which affects their performance in their role and results in a lack of guidance for continual improvement. Furthermore, weak communication infrastructure, particularly poor mobile networks and limited airtime, hinders referrals and coordination. Afari et al. (2014) and Getachew et al. (2021) both confirmed that communication gaps undermine service efficiency and the timely flow of health information.

Despite these barriers, the study found clear evidence of positive changes in the communities served by HEWs. Participants reported increased health awareness regarding sanitation, hygiene and malaria prevention, which is consistent with Banteyerga (2011), who showed that health education has significantly reduced preventable diseases. Recent studies by Mbata et al. (2024) have also confirmed that education leads to improved compliance with preventive measures and a reduction in malaria and diarrhoeal diseases. Community engagement has also strengthened, particularly through community adherence groups (CAGs) that support HIV treatment adherence. This aligns with Owiti (2024) and Panaite et al. (2024), who emphasised that peer-led support improves adherence and reduces stigma, strengthening community cohesion. Improvements have also been observed in maternal and child health, including a higher uptake of antenatal care, increased clinic deliveries and higher vaccination rates. These findings are supported by Anyanwu et al. (2024) and Olaboye et al. (2024), who confirmed that health education fosters safer maternal practices and promotes child health.

The participants proposed several recommendations to strengthen the HEWP, including career advancement opportunities, study leave and promotion pathways to enhance motivation. These findings are consistent with the work of Balushi et al. (2022) and Wai et al. (2024), who demonstrated that professional development is crucial for sustaining commitment among community health workers. Regular training and refresher courses were also emphasised, echoing Willie (2025) and Mohammed (2024), who noted that continuous education strengthens both competence and job satisfaction. The provision of educational and communication materials, such as mobile phones, airtime, and culturally relevant health leaflets, is another priority, reflecting findings from Hoxha et al. (2024). Participants also emphasised the importance of structured and supportive supervision, as recommended by Tesema et al. (2022) and Teshome et al. (2022), who have linked effective oversight to service quality improvements.

Finally, ensuring an adequate supply of medicines and other supplies, as well as improving occupational safety, emerged as key recommendations. This is consistent with Yirsaw et al. (2025), who reported that updated resources improve service effectiveness, and Jaskiewicz and Tulenko (2012), who noted that reliable supplies enhance productivity. Calls for protective clothing and transport support align with Nankongnab et al. (2021) and Jaca et al. (2022), who emphasised occupational safety and transport access as critical to effective care. Participants also stressed the importance of strengthening both community involvement and psychosocial support for HEWs. This is consistent with Russell et al. (2023), who highlighted community engagement and mental health support as being essential for building trust and improving service delivery.

### Strengths, limitations and areas for further research

The strengths of this study include that the participants were interviewed at times that were convenient for them, allowing them to share their experiences openly. The study provided detailed insights into the factors affecting the implementation of the HEWP, including challenges related to training, supervision, supplies and environmental conditions. The study was conducted in a limited number of communities, so the findings cannot be generalised to all HEWs or regions in Namibia. The study identified areas that require further investigation, including exploring the experiences of HEWs regarding the effectiveness of supervision, availability and management of essential resources, and community engagement strategies. Further research could also examine the long-term impact of environmental protection measures and training programmes on HEWs’ performance and retention.

### Recommendations

Based on the findings of this study, several recommendations are proposed to improve the effectiveness and implementation of the HEWP:

### Strengthening health extension workers’ professional development and skills

The study revealed gaps in knowledge, training and career advancement opportunities among HEWs. It is thus recommended that continuous professional development programmes, including refresher courses, workshops and in-service training, be implemented to enhance the knowledge, skills and confidence of HEWs. Opportunities for further studies, promotions and career progression should also be prioritised to improve motivation and job satisfaction.

### Provision of adequate resources and educational materials

Participants reported frequent shortages of essential resources, including referral forms, reporting tools, medicines and educational materials. It is recommended that the MoHSS and district authorities ensure a consistent and adequate supply of these resources. Additionally, HEWs should be provided with communication tools, such as mobile phones with network coverage and a monthly airtime allowance, to facilitate timely communication and effective health education in communities.

### Strengthening supervision and managerial support

The study found that irregular and unsupportive supervision limits the professional growth and performance of HEWs. It is recommended that a structured and consistent supervisory system be implemented, with supervisors specifically assigned to the HEWP. Regular supportive supervision will enhance accountability, improve service quality, and provide guidance in the field.

### Improving transportation and working conditions

Transportation challenges and unsafe working environments were identified as major barriers. It is recommended that adequate transportation support, such as vehicles or motorcycles, be provided for long-distance travel. Protective clothing, including uniforms, raincoats, gumboots, gloves and umbrellas, should also be provided to ensure the safety of HEWs in harsh environmental conditions.

### Enhancing community engagement and collaboration

Participants highlighted the importance of community support in improving healthcare delivery. It is therefore recommended that efforts be made to strengthen collaboration between HEWs and community members through health education, CAGs and mental health support initiatives. Assigning personnel to address social issues and ensuring timely follow-up of cases will enhance trust, engagement and the overall effectiveness of the HEWP.

## Conclusion

The study revealed that several factors influence the implementation of the HEWP, including challenges related to training and capacity, motivation and recognition, supportive supervision and feedback mechanisms, availability of essential materials and medications, environmental protection, and community engagement. The HEWs reported that inadequate resources, a lack of consistent supervision, and difficult working conditions hinder their ability to deliver timely and effective healthcare services. It is imperative that the MoHSS and relevant stakeholders address these factors to strengthen the HEWP. The outcomes of this study will contribute to improving healthcare service delivery, enhancing the effectiveness of health promotion activities, and ultimately leading to better health outcomes in communities. The results of this study can be used to develop targeted interventions and strategies to mitigate the factors encountered during the implementation of the HEWP in Namibia and similar settings.
